# Memory for Semantically Related and Unrelated Declarative Information: The Benefit of Sleep, the Cost of Wake

**DOI:** 10.1371/journal.pone.0033079

**Published:** 2012-03-22

**Authors:** Jessica D. Payne, Matthew A. Tucker, Jeffrey M. Ellenbogen, Erin J. Wamsley, Matthew P. Walker, Daniel L. Schacter, Robert Stickgold

**Affiliations:** 1 Department of Psychology, University of Notre Dame, Notre Dame, Indiana, United States of America; 2 Department of Psychiatry, Harvard Medical School, Boston, Massachusetts, United States of America; 3 Center for Sleep and Cognition, Boston, Massachusetts, United States of America; 4 Sleep Division, Department of Neurology, Massachusetts General Hospital, Boston, Massachusetts, United States of America; 5 Division of Sleep Medicine, Harvard Medical School, Boston, Massachusetts, United States of America States of America; 6 Department of Psychology, University of California, Berkeley, California, United States of America; 7 Department of Psychology, Harvard University, Cambridge, Massachusetts, United States of America; Catholic University of Sacred Heart of Rome, Italy

## Abstract

Numerous studies have examined sleep's influence on a range of hippocampus-dependent declarative memory tasks, from text learning to spatial navigation. In this study, we examined the impact of sleep, wake, and time-of-day influences on the processing of declarative information with strong semantic links (semantically related word pairs) and information requiring the formation of novel associations (unrelated word pairs). Participants encoded a set of related or unrelated word pairs at either 9am or 9pm, and were then tested after an interval of 30 min, 12 hr, or 24 hr. The time of day at which subjects were trained had no effect on training performance or initial memory of either word pair type. At 12 hr retest, memory overall was superior following a night of sleep compared to a day of wakefulness. However, this performance difference was a result of a pronounced deterioration in memory for unrelated word pairs across wake; there was no sleep-wake difference for related word pairs. At 24 hr retest, with all subjects having received both a full night of sleep and a full day of wakefulness, we found that memory was superior when sleep occurred shortly after learning rather than following a full day of wakefulness. Lastly, we present evidence that the rate of deterioration across wakefulness was significantly diminished when a night of sleep preceded the wake period compared to when no sleep preceded wake, suggesting that sleep served to stabilize the memories against the deleterious effects of subsequent wakefulness. Overall, our results demonstrate that 1) the impact of 12 hr of waking interference on memory retention is strongly determined by word-pair type, 2) sleep is most beneficial to memory 24 hr later if it occurs shortly after learning, and 3) sleep does in fact stabilize declarative memories, diminishing the negative impact of subsequent wakefulness.

## Introduction

The discovery that sleep benefits the retention of human memories dates back to Ebbinghaus [1], who inaugurated contemporary memory research with his seminal study of forgetting. He found that memory for nonsense syllables deteriorates rapidly at first, but progressively more slowly over the following 31 days. Two points on this monotonically decreasing memory curve did not seem credible to Ebbinghaus – 8.8 hours and 1 day – because while only 2.1% of information was forgotten across this 15.2 hour interval, three times that amount was forgotten across the next 24 hours. Ebbinghaus (1885, pp. 104–105) noted that much of the earlier 15-hour interval was occupied by sleep, but rejected this as an unlikely explanation of his findings.

Forty years later, Jenkins and Dallenbach [2] experimentally confirmed that memory retention following sleep was superior to retention following an equivalent period of wakefulness. Numerous studies have since replicated this ‘sleep effect’ employing an array of research designs and memory tasks [3], [4]. However, debate continues about the extent to which sleep-specific processes are responsible for these memory differences. Jenkins and Dallenbach, for example, concluded that forgetting in their study was “a matter of the interference, inhibition, or obliteration of the old by the new (p. 612)”, with sleep insulating memories from the disruptive influence of wakefulness. By this account, sleep transiently protects memories from retroactive interference, but only until they are exposed to interference the subsequent day [5]. Recently, an “opportunistic theory” of memory consolidation has been posited [6], which argues that *any* condition resulting in reduced exposure to interference will benefit declarative memory consolidation. Thus, sleep per se is not uniquely beneficial to memory.

Although protection from interference likely plays a role in the sleep effect, too few studies have employed research designs capable of parsing the unique contributions of sleep and wakefulness to memory processing, with many studies examining performance after a single 12 hr training-retest interval containing just one night of sleep vs. one day of wakefulness. Unfortunately, this 12 hr design does not clarify whether sleep benefits memory, or wakefulness impairs memory. However, memory performance following the first 12 hr interval containing a night of sleep or day of wakefulness can be compared to changes in memory that occur during the second 12 hr interval that also either contains a day of wakefulness or a night of sleep. This comparison allows for a more conclusive interpretation regarding the functional role of sleep and wakefulness on memory processing. Using this protocol, we were able to answer the following questions: Does sleep in the first 12 hr interval following training stabilize declarative memories, such that subsequent wakefulness has a diminished negative effect on the memory? Do people have to sleep shortly after task acquisition to experience the memory benefits of sleep, or does sleep benefit memory even when the sleep period does not begin until many hours (∼16 hr) after training? These questions are essential to answer in order to understand whether sleep has a lasting influence on declarative memory, and if so, how to best maximize sleep's benefit by sleeping at the appropriate time after learning.

The few studies that have tested memory at time points beyond an initial night of sleep have produced mixed results. For example, Benson & Feinberg (1975, 1977), using 8, 16, and 24 hr training-retest intervals, found that participants who slept soon after learning a list of unrelated paired associates retained more information 24 hours later than participants who endured a full day of waking activities prior to sleeping, while participants trained instead on nonsense syllables showed no difference between conditions [7], [8]. More recently, this delayed testing procedure was used to study learning of English-German vocabulary lists in high school students [9]. Participants learned word lists in the morning (8am) or evening (8pm) and were retested after 24 or 36 hr. Participants who learned the lists at 8pm, and who slept soon after training, showed enhanced recall relative to participants who learned the lists at 8am and experienced a full day of wakefulness prior to sleep. This suggests that a night of sleep shortly after encoding benefits performance. This “sleep-first” effect has also been shown with a task examining memory for face-location pairs [10], as well as with an observational learning task [11]. Interestingly, in one study with younger subjects (9–12 years old) immediate sleep and delayed sleep had a similar impact on word pair memory [12], suggesting this effect may be modulated by age.

While many studies have examined sleep's influence on hippocampus-dependent, declarative memories, there has yet to be a careful examination of precisely which types of declarative information are influenced by sleep. In fact, there is considerable debate about the extent to which semantically related and unrelated paired associates benefit from sleep [13], [14], and virtually nothing is known about the comparative impact of wakefulness on these two forms of declarative memory. While some studies report a post-sleep recall advantage for word pairs that have strong pre-existing semantic relationships (*e.g.*, circus – clown; [15], [16]), others suggest that sleep benefits the processing of word pairs that *lack* a pre-established semantic relationship (*e.g.*, cactus – brick; [7], [8]).

Although several previous sleep studies have examined performance on these two word pair types independently, here we directly compared memory for the two types of word pairs – those with a well-established semantic relationship, and those lacking a semantic relationship and therefore requiring the creation of novel associations between items in each word pair. By training participants at either 9am or 9pm and then retesting them at 30 min, 12 hr, or 24 hr, we were able, for the first time, to fully clarify the roles of sleep, wake, and time-of-day influences on memory for these different forms of declarative memory. An additional benefit of testing both types of memory is that it permits us to draw broader and stronger conclusions about the roles of sleep and wake on hippocampus-dependent memory consolidation.

## Methods

### Ethics Statement

Written informed consent was obtained from all participants, and the study was approved by the Beth Israel Deaconess Medical Center Institutional Review Board at Harvard Medical School.

### Participants

Participants were 207 healthy, medication free Harvard students, assigned to three experimental conditions – *30 min*: N = 60 (31 evening, 29 morning), 37 females, mean age = 20.6; *12 hr*: N = 80 (40 Sleep, 40 Wake), 52 females, mean age = 20.3; *24 hr*: N = 67 (33 Immediate Sleep, 34 Delayed Sleep, 40 females, mean age = 20.3). Participants had no prior history of mood or sleep disorders, and habitually slept at least 6 hours per night. They were instructed to sleep for at least 6 hours on the night prior to the experiment and not to take naps on the day of the experiment. Caffeine and alcohol were prohibited for 24 hr prior to and throughout the study. These criteria were confirmed by questionnaire on the day of participation. Participants received payment or course credit for their participation.

### Design and procedure

**Figure 1:**
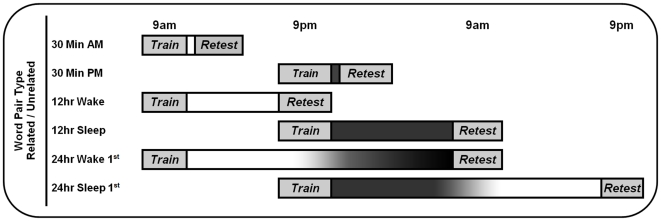
Study Design. White indicates wakefulness; dark gray indicates sleep.

Participants were randomly assigned to study semantically related or unrelated word pairs at 9am or 9pm (see Figure 1 for study design). Upon arriving in the laboratory, participants gave written informed consent and completed the Stanford Sleepiness Scale [17] and a sleep log on which they recorded bed times and wake times for the previous two days. Participants then sat at computers and were trained on the word pairs to a learning criterion of 60% (see below). Once this criterion was reached, participants left the laboratory, and returned for testing 30 min, 12 hr, or 24 hr later depending on experimental condition. Upon their return, participants sat at the same computers and completed the Stanford Sleepiness Scale a second time prior to retest.

### Memory task and materials

We used the two lists of semantically related word pairs that were previously translated into English by Gais & Born (2004) (Appendix S1, Lists 1 and 2; [18]). These pairs are equated for concreteness, emotionality, imagery, meaningfulness, potency, and valence. To create two lists of unrelated pairs, we took the cue words from Lists 1 and 2 and randomly paired them with other target words from the lists. Pairs that still appeared related were randomized again, until two lists of unrelated word pairs were created (Appendix S2, Lists 3 and 4). Participants were pseudorandomly assigned to one of the four lists.

Prior to training on the word pairs, participants were read scripted instructions. They were told to learn each of the 40 word pairs to the best of their ability because their memory would be tested until they were able to recall 24 of the 40 correct responses (60% criterion). Word pairs were presented sequentially on a computer screen for 5 sec each, with an inter-trial interval of 1000 ms. The order of presentation of the word pairs was randomly determined for each participant. Following presentation of the pairs, participants performed a cued recall test in which the first word of each pair was presented (again in random order), and participants were instructed to type in the word that completed the pair. After each response, the correct pair was displayed for 2 seconds, allowing participants to relearn the pair if necessary. If participants failed to successfully recall 24 correct responses, word pair order was again randomized and the cued recall test (with feedback) was repeated until the 60% criterion was reached.

Note that feedback on the final training trial constitutes a learning event that does not manifest itself in performance until the retest session; thus, performance will tend to increase between the end of training and the final retest. At retest 30 min, 12 hr, or 24 hr later, participants were tested one final time with no feedback. Memory performance was measured as the change in recall from training to retest (% Correct at Retest – % Correct at Training).

## Results

### 30 Minute Retest

Participants in the Morning (9am) and Evening (9pm) groups reached criterion after a similar number of training trials and achieved similar scores on the final training trial (Table 1). Stanford Sleepiness Scale ratings did not differ between groups, either at training (am = 2.79 vs. pm = 2.73, p = .89) or retest (am = 2.21 vs. pm = 2.67, p = .12).

**Table 1:** Performance at training on unrelated and related paired-associates in the 30 minute groups (Morning, Evening).

		*Retention Interval*			
	*Response Measure*	*30 min AM*	*30 min PM*	t	p
	Trials to Criterion	2.8 (0.4)	2.5 (0.3)	0.84	0.41
Unrelated	% Correctly Recalled on Criterion Trial	71.1 (1.8)	70.2 (1.9)	0.37	0.71
	Trials to Criterion	1.4 (0.2)	1.4 (0.2)	0.23	0.82
Related	% Correctly Recalled on Criterion Trial	75.6 (3.0)	75.2 (2.8	0.11	0.91

A 2×2 ANOVA with Condition (Morning, Evening) and Word-pair type (Related, Unrelated) as between-subjects factors revealed no effect of Word-pair type (F_1,56_ = 0.20, p = .89) or Condition (F_1,56_ = 1.05, p = .31), nor a Condition×Word-pair type interaction (F_1,56_ = 0.47, p = .50). This result indicates that performance was not influenced by time of task encoding (Morning vs. Evening), and that initial memory performance at 30 min was the same regardless of word pair type.

### 12-Hour Retest

There were no performance differences between the 12 hr groups at the end of training, as measured by the number of trials required to reach criterion or the percent correct responses during the final training trial (Table 2). Stanford Sleepiness Scale ratings also did not differ between Sleep and Wake participants, either at training (2.77 vs. 2.79, p = .94) or at retest (2.41 vs. 2.79, p = .24).

**Table 2:** Performance at training on unrelated and related paired-associates in the 12 hour groups (Wake, Sleep).

		*Retention Interval*			
	*Response Measure*	*Wake*	*Sleep*	t	p
Unrelated	Trials to Criterion	3.0 (0.3)	2.9 (0.3)	0.22	0.83
	% Correctly Recalled on Criterion Trial	68.6 (1.6)	70.9 (1.9)	0.96	0.34
Related	Trials to Criterion	1.4 (0.1)	1.6 (0.1)	1.2	0.26
	% Correctly Recalled on Criterion Trial	75.3 (2.1)	78.6 (1.7)	1.3	0.21

**Figure 2:**
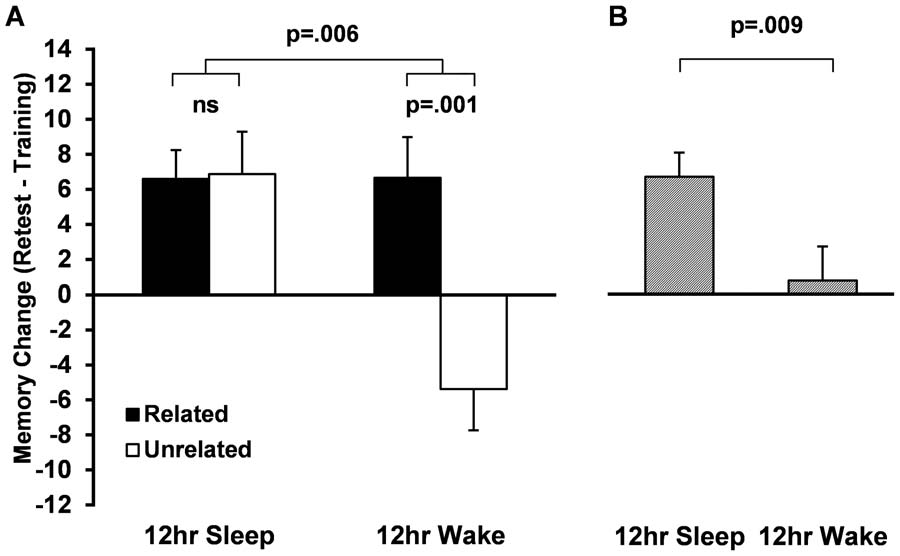
12 hr Performance Data. A. Change in recall from training to retest for Related (black bars) and Unrelated (white bars) word pairs in the 12 hr Sleep and Wake groups. B. Data collapsed across word pair type (striped bars). Bars represent % recall at training minus % recall at retest (means±SEMs).

Looking at performance at the 12 hr retest, a 2 (Condition, Sleep vs. Wake)×2 (Word-pair type, Related vs. Unrelated) ANOVA revealed a significant main effect of Condition (change from training: 12 hr Sleep: +6.6%, 12 hr Wake: +0.8%, F_1,76_ = 7.23, p = .009; Figure 2b), indicating that word pair recall was superior 12 hr later when the retention interval spanned a night of sleep, rather than a day of wakefulness. The overall advantage in the 12 hr Sleep group was qualified by a significant Condition×Word-pair type interaction (F_1,76_ = 7.94, p = .006; Figure 2a). Several findings are of interest here. First, while wake and sleep led to equivalent recall of related word pairs (t_40_ = 0. 01, p = .92), recall of unrelated pairs was disrupted in the wake relative to the sleep condition (t_36_ = 3.62, p = .001, white bars). Second, although recall of related and unrelated pairs was equivalent in the sleep condition (t_38_ = 0.03, p = .98, Figure 2a), there was a significant difference between recall of related and unrelated pairs in the wake condition (t_38_ = 3.63, p = .001).

### 24 Hour Retest

Table 3 summarizes word-pair recall for related and unrelated pairs at encoding. As in the 30 min and 12 hr conditions, there were no group differences at training indicated either by the number of trials required to reach criterion or the percent correct responses during the final training trial. Stanford Sleepiness Scale ratings did not differ between the 24 hr Delayed and 24 hr Immediate sleep groups at training (2.79 vs. 2.64, p = .53) or retest (2.46 vs. 2.53, p = .81).

**Table 3:** Performance at training on unrelated and related paired-associates in the 24 hour groups (24 hr am, “Delayed sleep”; 24 hr pm, “Immediate sleep”).

		*Retention Interval*			
	*Response Measure*	*24 hrImmediate Sleep*	*24 hr Delayed Sleep*	t	p
Unrelated	Trials to Criterion	2.6 (0.3)	3.3 (0.3)	1.6	0.13
	% Correctly Recalled on Criterion Trial	73.7 (2.1)	70.9 (1.7)	1.1	0.29
Related	Trials to Criterion	1.5 (0.1)	1.4 (0.1)	0.56	0.58
	% Correctly Recalled on Criterion Trial	76.9 (2.5)	75.6 (1.8)	0.43	0.67

*Note:* Values are means with SEM in parentheses. Note that across all three experiments, training in the morning (*Wake, 30 min am and 24 hr am groups*) did not differ from training in the evening (*Sleep, 30 min pm and 24 hr pm groups*) on the number of trials it took to reach the 60% criterion or % correct responses on the final (i.e. criterion) training trial.

**Figure 3:**
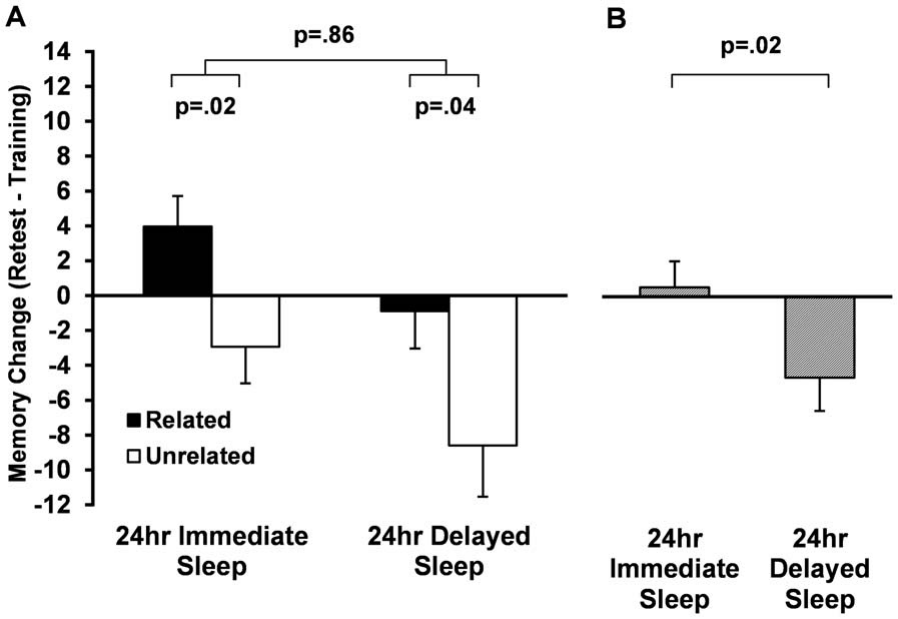
24 hr Performance Data. A. Change in recall from training to retest for Related (black bars) and Unrelated (white bars) word pairs in the 24 hr Immediate Sleep and Delayed Sleep groups. B. Data collapsed across word pair type (striped bars). Bars represent % recall at training minus % recall at retest (means±SEMs).

As with the 12 hr retest condition, there was a main effect of Condition (F_1,63_ = 5.44, p = .02; Figure 3b) demonstrating that participants in the Immediate Sleep Condition performed much better than the Delayed Sleep Condition at retest (change from training: 0.7±2.1% vs. −6.5±2.6%). Thus, subjects benefited from having sleep come in the first, rather than second, 12 hr interval. There was also a significant effect of Word-pair type (F_1,63_ = 10.5, p = .002), but no interaction between Condition and Word-pair type (F_1,63_ = 0.03, p = .86; Figure 3a). Thus, 24 hours after learning, the difference in recall between related and unrelated word pairs was similar in the Immediate and Delayed conditions (6.9±2.8% and 7.7±3.6%, respectively), and significant in each condition (Immediate Sleep: p = .02; Delayed Sleep: p = .04; Figure 3a).

### 12 h v. 24 hr Comparisons

**Figure 4:**
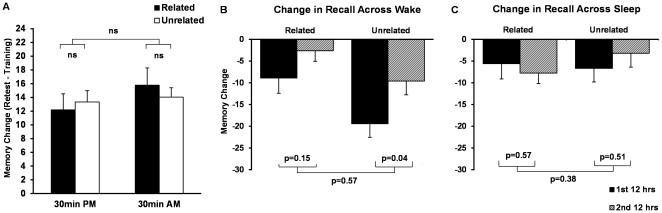
Memory Across the First and Second 12 hr Intervals Containing either Wake or Sleep. A. Change in recall from training to retest for Related (black bars) and Unrelated (white bars) word pairs in the 30 min groups. Bars represent % recall at training minus % recall at retest (means±SEMs). B. Memory (% correct) following the first and second 12 hr interval of Wake was measured as the difference following 12 hr Wake minus 30 min Morning recall (black bars) and 24 hr Immediate Sleep minus 12 hr Sleep (striped bars). C. Memory (% correct) following the first and second 12 hr interval of Sleep – 12 hr Sleep minus 30 min Evening (black bars); 24 hr Delayed Sleep minus 12 hr Wake (striped bars). Bars are means±SEMs.

To determine the extent of memory change across 12 hr of wake or sleep during the first 12 hr post-training, we used the 30 min data as a reference point (Figure 4a) to compare change in memory from 30 min to 12 hr retest. To assess memory across the second 12 hr interval, which contained wake or sleep, we compared change in memory from 12 hr to 24 hr retest. As one example (Figure 4b), by subtracting the amount of forgetting in the 12 hr Sleep groups from that seen in the 24 hr Immediate Sleep groups, we obtained a measure of forgetting during the second 12 hr (filled with wake) in the Immediate Sleep condition. Across 12 hr of wake (Figure 4b), substantially more forgetting was seen overall when the wake time came in the first as opposed to second 12 hr post-training (F = 5.6, p = 0.019). Post-hoc tests demonstrated twice as much forgetting of unrelated pairs during wake in the first 12 hr than in the second 12 hr post-training (p = .04), and 3.4 times more forgetting of related pairs, although this difference did not reach significance. Thus, a night of sleep appears to slow the rate of forgetting during subsequent wakefulness.

In contrast, forgetting across 12 hr intervals including a night of sleep (Figure 4c) averaged 5.8% of studied word pairs for both related and unrelated pairs across both the first and second 12 hr post-training, with no significant differences based on word pair type or whether the sleep came in the first or second 12 hr. Thus, deterioration in recall was unaffected by word pair type, and is similar regardless of whether the sleep comes immediately after training or only following 12 hr of post-training wake.

## Discussion

In this study we examined the impact of sleep, wake, and the time-of-day on memory for declarative information with strong semantic links (semantically related word pairs) and information requiring the formation of novel associations (semantically unrelated word pairs). By training participants on paired associates at 9am or 9pm, and then retesting them after a 30-minute, 12-hour, or 24-hour delay, we were able to more comprehensively assess memory when sleep and wake immediately followed training or occurred many hours after training.

As expected, across all conditions we observed similar training performance regardless of whether subjects trained in the morning or evening. Importantly, we also found that retention following a relatively brief 30 min interval was similar for subjects who trained in the morning or evening, and that there was no difference between recall of related and unrelated word pairs, suggesting that memory acquisition and retrieval after a relatively brief delay were not influenced by whether subjects performed the task in the morning or evening.

As expected, following a 12 hr retention interval containing a night of sleep or a day of wakefulness, overall recall was superior in subjects who slept. Interestingly, this is precisely what an opportunistic theory of memory consolidation would predict [5], [6]. However, this memory effect was strongly modulated by word pair type, with sleep and wake exerting a similar effect on related, but not unrelated, word pairs. Thus, while the broad pattern of results reported here appears to fit a more opportunistic account of memory consolidation, the dissociable and differential effects of wake and sleep on consolidation of related and unrelated information is not parsimoniously explained by such an account. Similar patterns of selective consolidation following sleep have been observed in several other domains of declarative memory, including emotional memory [19], [20], [21], [22], semantic memory [23], and direct encoding [24], [25], [26]. One particularly relevant study demonstrated preferential consolidation of abstract (vs. concrete) word-pairs that are more difficult to learn, a finding that was strongly dependent on sleep spindle activity [27]. To the extent that our unrelated word pairs were also more difficult to encode, one might predict that our 12 hr finding (see Figure 2) might depend on activity in the delta and spindle frequency ranges [27], [28], [29], [30], a hypothesis that should be explored using EEG recordings. Together, these studies demonstrate that exposure to interfering information during wakefulness is not equally disruptive to all forms of declarative memory, suggesting that such models remain incomplete in their characterization of sleep-based memory consolidation.

To better understand the mnemonic contribution sleep and wake make to these two word pair types, we assessed memory after a 24 hr interval, which contained equal amounts of sleep and wakefulness. The inclusion of the 24 hr conditions yielded two important findings that cannot be obtained by analysis of 12 hr data alone. First, we were able to assess the impact of sleep when it occurs shortly after training in the evening (24 hr Immediate Sleep condition), compared to when it comes after a full day of wakefulness, up to 16 hr following training in the morning (24 hr Delayed Sleep condition). Participants who slept immediately after learning recalled significantly more word pairs overall than those whose sleep was delayed for 16 hr post-encoding. This finding is in line with other studies showing that sleep has its most pronounced benefit for hippocampus-dependent memory within a few hours of encoding (e.g. Vocabulary learning [9]; Emotional memory [28]; Spatial memory [10]), which clearly has practical benefits for learning (*i.e.*, studying one final time for an exam prior to bedtime).

Second, we were able to assess the stabilizing effect of sleep on two forms of declarative memory (i.e. semantically related and unrelated word pairs) by comparing whether memory following a night of sleep (12 hr Sleep condition) is comparable to memory following 24 hr, when a night of sleep is followed by a full day of wakefulness. The 24 hr data suggest that sleep has a stabilizing effect on both forms of declarative memory; the rate of deterioration during an interval of wake that follows sleep is significantly slower than when sleep does not precede the wake period.

The idea that sleep benefits retention dates back to the pioneering work of Ebbinghaus (1885 [1]). Using nonsense syllables, Jenkins and Dallenbach [2] showed that while declarative memories tend to deteriorate across time, the deterioration was substantially less when participants slept. They concluded that “forgetting is not so much a matter of the decay of old impressions and associations as it is a matter of the interference, inhibition, or obliteration of the old by the new.” However, that study was not able conclusively to demonstrate whether it was sleep or wake processes that accounted for their results. Our results give a more complete understanding of the unique impact of sleep and wake on declarative memory. First, a 12 hr delay has a dramatically different impact on memory for related and unrelated word pairs depending on whether one sleeps or remains awake. Importantly, exposure to 12 hr of waking interference does not negatively impact memory for semantically related word pairs, but only for unrelated pairs, in spite of equivalent performance on the word pair types at the end of training. Second, with a longer delay it becomes apparent that sleep does have a beneficial effect on memory for both word pair types, but only if it comes shortly after encoding, not when delayed by 16 hr following encoding. Finally and most importantly, when sleep shortly follows declarative task learning, it actually slows the subsequent rate of deterioration during the post-sleep wake period, suggesting that an important function of sleep is to stabilize newly learned declarative memories. In line with a growing number of studies [31], [32], [33] these results suggest that sleep's benefit to memory cannot be easily explained simply by when participants learned the task, or by interference arguments alone.

## Supporting Information

Appendix S1 Related Word Pairs.
(DOC)

Appendix S2 Unrelated Word Pairs.
(DOC)
