# Supplementary material for: Memory for Semantically Related and Unrelated Declarative Information: The Benefit of Sleep, the Cost of Wake
Source: PLoS One. 2012 Mar 22;7(3):e33079. doi: 10.1371/journal.pone.0033079 (PMC3310860; doi:10.1371/journal.pone.0033079)
Supplement: Supplementary file 1 [file pone.0033079.s001.doc]

LIST 1

school	blackboard
tower	bell
sea	tide
family	marriage
newspaper	interview
sonata	joy
banner	camp
[illegible]dency	increment
mother	child
insect	caterpillar
[illegible]iver	ship
coast	beach
gun	bullet
blacksmith	metal
home	room
building	hall
rain	flood
avenue	tree
decency	truth
decree	decision
diamond	hardness
result	effect
occupation	doctor
book	story
attack	operation
cat	soul
doll	cradle
episode	happiness
railroad	steam
kitchen	pot
countryside	swamp
musician	pianist
industry	factory
clothing	scarf
car	headlight
prison	gangster
gale	wind
bouquet	blossom
bottle	toast
[illegible]	person
crisis	emergency
[illegible]	engagement
harbor	crane
garden	flowerbed

LIST 2

rider	switch
fire	smoke
animal	fox
road	car
weaver	troubles
flakes	rescue
rein	turn
mission	messenger
furniture	chair
body	blood
army	admiral
friend	loyalty
bird	lark
celebration	alcohol
reptile	frog
grain	oats
joint	knuckle
artist	painting
statement	doubt
revolt	policeman
alliance	betrayal
event	incident
factory	foreman
plant	leaf
tenant	rent
commercial	candy
giant	club
trip	map
mountain	boulder
ruler	palace
play	drama
illness	doctor
church	heaven
infection	bacteria
university	semester
underworld	crime
instrument	bagpipes
glacier	avalanche
idea	proverb
faith	renouncement
theory	concept
authority	state
fir	needle
circus	clown
