# Supplementary material for: Memory for Semantically Related and Unrelated Declarative Information: The Benefit of Sleep, the Cost of Wake
Source: PLoS One. 2012 Mar 22;7(3):e33079. doi: 10.1371/journal.pone.0033079 (PMC3310860; doi:10.1371/journal.pone.0033079)
Supplement: Supplementary file 2 [file pone.0033079.s002.doc]

LIST 3

school	crane
tower	flowerbed
sea	bullet
family	metal
newspaper	operation
sonata	increment
banner	flood
[illegible]dency	camp
mother	steam
insect	truth
[illegible]iver	hall
coast	soul
gun	tide
blacksmith	tree
home	scarf
building	child
rain	marriage
avenue	joy
decency	factory
decree	room
diamond	ship
result	gangster
occupation	emergency
book	cradle
attack	happiness
cat	toast
doll	beach
episode	person
railroad	interview
kitchen	effect
countryside	decision
musician	engagement
industry	doctor
clothing	story
car	caterpillar
prison	pianist
gale	hardness
bouquet	headlight
bottle	swamp
	wind
crisis	blossom
	pot
harbor	blackboard
garden	bell

LIST 4

rider	needle
fire	clown
animal	incident
road	[illegible]
weaver	blood
flakes	chair
rein	frog
mission	boulder
furniture	loyalty
body	semester
army	concept
friend	palace
bird	troubles
celebration	avalanche
reptile	map
grain	policeman
joint	fox
artist	state
statement	rescue
revolt	bagpipes
alliance	bacteria
event	doctor
factory	doubt
plant	admiral
tenant	drama
commercial	knuckle
giant	heaven
trip	crime
mountain	alcohol
ruler	leaf
play	oats
illness	lark
church	turn
infection	car
university	renouncement
underworld	proverb
instrument	foreman
glacier	messenger
idea	rent
faith	club
theory	betrayal
authority	painting
fir	smoke
circus	switch
